# Characterization of local linearity in deep learning CT denoising networks

**DOI:** 10.1002/mp.70756

**Published:** 2026-11-05

**Authors:** Kwok Lung Fan, Junyuan Li, Craig Abbey, Cheng Ting Lin, J. Webster Stayman, Grace J. Gang

**Affiliations:** ^1^ Department of Radiology University of Pennsylvania Philadelphia Pennsylvania USA; ^2^ Department of Medical Physics Memorial Sloan Kettering Cancer Center New York New York USA; ^3^ Department of Biomedical Engineering Johns Hopkins University Baltimore Maryland USA; ^4^ Department of Psychological and Brain Sciences University of California Santa Barbara Santa Barbara California USA; ^5^ Department of Radiology Johns Hopkins University School of Medicine Baltimore Maryland USA

**Keywords:** CT denoising, Deep learning evaluation, Image quality, Jacobian analysis, Local linearity, Neural Network interpretability

## Abstract

**Background:**

Deep learning algorithms for image processing have been proposed for a wide range of applications. In particular, deep learning CT denoising has demonstrated superior ability to reduce noise compared to conventional methods. However, the nonlinear nature of these algorithms makes it difficult to inspect and visualize how the model accomplishes the denoising task.

**Purpose:**

We propose a local linearization approach to characterize and analyze the behavior of deep learning algorithms. Example analysis was performed for a classic denoising architecture with convolutional neural network and ReLU activation functions, and was extended to GELU activation and a transformer‐based architecture.

**Methods:**

We defined strictly local linear regions as the input space over which the activation patterns of all ReLU activation functions are the same. The extent of these regions was investigated by perturbing clinically relevant features of a lung lesion. We further defined approximately local linear regions as the input space where Jacobians are similar and linear approximation results in small error. We developed an algorithm to partition input space over a range of lesion and background features into approximately local linear regions, and separately quantified the contribution of the noise realization and anatomical background to this approximation error. The behavior of networks trained with different levels of denoising, as well as alternative architectures, was compared.

**Results:**

The extent of strictly local linear regions was found to be very small. Perturbations on the scale of $10^{-8}{\text{mm}}^{-1}$ were enough to push the network to a neighboring strictly local linear region. However, the Jacobians over a large number of strictly local linear regions were found to be highly similar, which suggests that the network behaves approximately as a piecewise linear system. The partitioning algorithm identified a small number of approximately local linear regions within input spaces characterized by clinically relevant features; this number was similar for the GELU variant but increased for networks trained with more aggressive noise reduction and for a transformer‐based architecture, while Jacobians transferred across anatomical backgrounds also increased approximation error.

**Conclusions:**

The local linearity analysis framework can be a useful tool for elucidating the behavior of neural networks that are traditionally considered as “black‐boxes.” Preliminary results suggest that the framework can be applied to other differentiable architectures, subject to empirical validation, to characterize these highly nonlinear, data‐dependent algorithms over input spaces of interest.

## 1 INTRODUCTION

In recent years, deep learning has made significant strides in medical imaging, enabling new capabilities across a range of tasks.^1^ In computed tomography (CT), many studies have proposed deep learning image reconstruction and/or post‐processing for potential improvements in diagnostic performance. A variety of network architectures have been adopted and produced visually pleasing images with high spatial resolution, low noise, and minimal artifacts. However, the nonlinear and data‐dependent nature of these models poses challenges for image quality evaluations. It is often unclear how to systematically characterize or interpret their behavior. Concerns have also been raised when key diagnostic features such as lesion contrast or size are misrepresented, or when false features that look realistic are created.^2^

Traditional image quality (IQ) metrics such as the modulation transfer function (MTF) and noise power spectrum (NPS) have long been used to characterize linear or linearized imaging systems.^3^ Most efforts to evaluate deep learning (DL) models still rely on these traditional metrics, typically applied to specific phantoms or datasets.^4^ However, these methods are grounded in linear systems theory, assuming linear, shift‐invariant system response and stationary noise. While appropriate for conventional filtered backprojection reconstruction or post‐processing using linear filters, these assumptions no longer hold in DL models, which are nonlinear and can be strongly data dependent. As a result, standard IQ metrics may fail to capture important behaviors of DL processing, especially in cases involving realistic anatomical features. Furthermore, they do little to explain why a network behaves the way it does.

Local linearization provides a principled framework for analyzing nonlinear systems and has been increasingly applied to deep learning models in medical imaging.^5^, ^6^ Importantly, convolutional neural networks (CNNs) with rectified linear unit (ReLU) activations are piecewise linear by construction. For any fixed activation pattern across all ReLU units, the network behaves as an exact linear operator. Transitions between activation patterns partition the input space into locally linear regions. While this structural property is well known, the practical question remains: over what regions of clinically relevant input space does approximate linearity hold, and how many distinct local linear regions are required to describe network behavior? Prior studies have leveraged first‐order Taylor expansion to analyze noise and resolution characteristics of CNNs. For example, Guo et al.^7^ modeled noise propagation using Jacobian‐based linearization, and Xu and Noo^8^ developed a Fourier‐domain framework to estimate resolution and noise properties efficiently without Monte Carlo simulation. These works demonstrate that linear systems tools can be applied locally to CNN‐based reconstruction and denoising models, but they do not explicitly characterize the structure and extent of the piecewise‐linear regions in clinically meaningful feature spaces.

In this work, we systematically characterize the extent of local linearity for a CNN‐based CT denoising network by explicitly mapping the input space into approximately local linear regions under a predefined approximation threshold. Crucially, the proposed framework is designed as a diagnostic and analytical tool intended specifically to characterize the mathematical behavior of a pre‐trained network, rather than serving as an independent reconstruction algorithm or analyzing variations introduced during model training. Building on prior Jacobian‐based analyses,^9^, ^10^, ^11^ which focused on local system response characterization, noise prediction, and visualization of preserved and attenuated perturbation subspaces, we extend the framework to quantify how many operating points are required to approximate the model response across multi‐dimensional clinical‐relevant parameters such as contrast, lesion size, spiculation, and background intensity. Rather than analyzing a single operating point, we investigate the global organization of local linear regions and evaluate the contribution of the noise realization as well as the stability of these regions across a range of clinically relevant regions of the input space. This analysis provides an interpretable bridge between nonlinear deep learning models and classical linear systems theory, enabling more systematic characterization of model behavior and potential failure modes.

## 2 METHODS

During the preparation of this manuscript, the authors used Claude (Anthropic) Opus 5.5 and Sonnet 5 solely for language editing, including grammar checking and proofreading of the text. All scientific content, analyses, and conclusions were produced by the authors, who reviewed and take full responsibility for the final manuscript.

### 2.1 Theoretical methods

Convolutional neural networks typically consist of linear layers (e.g., convolutional layers and fully connected layers) and nonlinear activation layers like the Rectified Linear Unit (ReLU), sigmoid function, and leaky ReLU, usually in an alternating arrangement. In some models, the only nonlinearity comes from the activation layers, making them strictly linear in the case of small‐scale local perturbations.^9^ In this work, we focus our discussion on the popular ReLU activation function, but a similar linear approximation can be applied to other activation functions as long as the change in input is small.

The use of the ReLU function in machine learning was proposed as an alternative activation^12^, ^13^ to the sigmoid function and has become the most commonly used activation function in neural networks. The ReLU function is a piecewise linear function defined as:

(1) ReLU(x)=max(0,x),

where x is the function input. For each ReLU, we can further define the *activation indicator*, o as

(2) $$o=\left\{\begin{array}{cc} 1 & \text{if ReLU}(x)>0\\ 0 & \text{otherwise}. \end{array}\right.$$

For a trained model, the output is governed by the weights of the linear layers and the ReLU indicators. Following Raghu et al.^14^, we define the set of all activation indicators in the network as an activation pattern O:

(3) $$O=\{o^{1},o^{2},\text{…},o^{N}\},$$

where N is the total number of ReLU units in the network. Within the same activation pattern, the input of the network is linearly related to the output. Therefore, we can express the network output $\mu_{k}$ as the following layer‐wise cascaded system:

(4) $$\mu_{k}=H\left(\mu_{o}\right)=h_{L}\circ \cdots \circ h_{2}\circ h_{1}\left(\mu_{o}\right),$$

where the operator ∘ denotes function composition. The l‐th layer, $h_{l}$, is defined as

(5) $$h_{l}\left(\mu_{l-1}\right)=O_{l}\left(W_{l}\mu_{l-1}+b_{l}\right),$$

corresponding to the convolution or linear layer l in the network. The weight matrix and bias vector of layer l are denoted by $W_{l}$ and $b_{l}$, respectively. The matrix $O_{l}$ is a diagonal matrix whose diagonal elements indicate the activation status of each unit.

#### 2.1.1 Strictly local linear regions

In theory, the network within each activation pattern is a linear system, and the response to all inputs triggering the same activation pattern can be characterized using the same system response. We define this input space as the **strictly local linear region**. We can compute the Jacobian matrix at a given input in this region (also referred to as the operating point) $\mu_{o}$ and obtain the output for any other inputs within the region according to:

(6) $$H\left(\mu_{k}\right)=H\left(\mu_{o}\right)+J_{o}\left(\mu_{k}-\mu_{o}\right).$$

where the Jacobian, $J_{o}$, is the first‐order derivative of the system output with respect to the input

(7) $$J_{o,ij}=\frac{\partial H{\left(\mu_{o}\right)}_{i}}{\partial \mu_{o,j}},$$

where i and j are the indices of the input. In matrix form, the full Jacobian can be written as

(8) $$J_{\mu_{o}}=\left[\begin{array}{ccc} \frac{\partial \mu_{k,1}}{\partial \mu_{o,1}} & \cdots & \frac{\partial \mu_{k,1}}{\partial \mu_{o,j}}\\ \vdots & \ddots & \vdots \\ \frac{\partial \mu_{k,i}}{\partial \mu_{o,1}} & \cdots & \frac{\partial \mu_{k,i}}{\partial \mu_{o,j}} \end{array}\right].$$

The entire network is effectively piecewise linear and can be characterized by the set of Jacobian matrices over the entire input space, with each Jacobian corresponding to a distinct activation pattern. The input space typically has a dimensionality equal to the number of voxels in the input image, with each dimension spanning the range of possible voxel values. Any evaluations over this input space, however, is computationally expensive. We therefore project the input space to specific image features that are of clinical interest, e.g., lesion contrast, lesion size, and perform subsequent investigations within the input space corresponding to a set of features.

To examine the extent of a strictly local linear region, we introduce small perturbations of increasing magnitude around a specific operating point and monitor the resulting changes in the network's activation patterns. During forward propagation, the binary activation states (active versus inactive) of all N ReLU units across all layers are recorded to form a complete network activation pattern, denoted as O.

To precisely capture transitions between successive steps, the cumulative number of activation‐state changes, C(k), up to perturbation step k is calculated by recursively incrementing the count from the preceding step:

(9) $$C\left(k\right)=C\left(k-1\right)+\sum_{i=1}^{N}\left|o_{i}\left(k\right)-o_{i}\left(k-1\right)\right|,$$

where C(0)=0 represents the signal‐absent baseline background. Expressed explicitly, the total cumulative number of changes at perturbation step k is given by:

(10) $$C\left(k\right)=\sum_{j=1}^{k}\sum_{i=1}^{N}\left|o_{i}\left(j\right)-o_{i}\left(j-1\right)\right|.$$

By tracking these activation patterns between consecutive, finely spaced perturbation steps, we can identify the boundaries of distinct locally linear regions. Within any single local region, the complete activation pattern remains identical across all units, manifesting as a distinct plateau or flat segment in the cumulative change profile. Because the step size between successive perturbation levels is minute, this incremental tracking allows us to map the precise boundaries of the local linear regions with high fidelity.

The extent of strictly local linear regions will be investigated for example inputs and perturbations in Section 2.2.3.

#### 2.1.2 Approximately local linear regions

The Jacobians for *neighboring* strictly local linear regions can be similar. Therefore, we define an **approximately local linear region** as a set of strictly local linear regions that have approximately the same system response. For all inputs within the approximately local linear region, their outputs can be approximated using the Jacobian at an operating point:

(11) $$H\left(\mu_{k}\right)\approx H\left(\mu_{o}\right)+J_{o}\left(\mu_{k}-\mu_{o}\right).$$

##### 2.1.2.1 Local linear approximation error

In a perfectly linear system, the Jacobian is constant across the input space. For our network, neighboring regions with similar Jacobians provide supporting evidence that the system behaves approximately linearly. Rather than comparing Jacobian matrices to one another directly, we quantify this similarity in the image domain, through the discrepancy between the true network output and the output predicted by the linear model built at the operating point. For a perturbation Δμ away from an operating point $\mu_{o}$, we define the **linear approximation error** as

(12) $$\varepsilon \left(\Delta \mu \right)=H\left(\mu_{o}+\Delta \mu \right)-\left[H\left(\mu_{o}\right)+J_{o}\;\Delta \mu \right],$$

where $J_{o}$ is the Jacobian evaluated at $\mu_{o}$. For thresholding, the error matrix is reduced to a scalar by averaging over a region of interest, either as an average absolute error or as an average per‐pixel percentage error. Either summary can serve as a threshold for determining the perturbation at which the linear approximation breaks down, and hence whether two strictly local linear regions belong to the same approximately local linear region. With a fixed error threshold, we can compute the maximum perturbation scale at which the error of the linear approximation remains below the threshold. Consequently, the size of each approximately local linear region, and therefore the number of operating points required to cover a given perturbation range, is threshold‐dependent. A stricter error threshold yields smaller regions and requires more operating points, whereas a more permissive threshold requires fewer. Given the perturbation type, range, and error threshold, we can potentially describe the system using just a few linear regions. This makes it possible to apply linear system analysis to the model.

##### 2.1.2.2 Partitioning algorithm

To characterize regions where the network behaves approximately linearly, we developed a greedy grid search algorithm to iteratively identify operating points and their associated approximately local linear regions within a pre‐defined feature input space (e.g., over clinically relevant ranges of lesion contrast and size). We first defined a fixed‐resolution grid over the input space. We placed the first operating point at the grid center, and computed its Jacobian. For each grid point, the approximation error of Equation (12) relative to the current set of operating points was evaluated.

All grid points with errors exceeding a predefined threshold were added to a stack. The grid point with the highest error was then selected from the stack as a new operating point, and its Jacobian was computed. The approximation errors for the remaining grid points in the stack were updated according to the lowest error over all existing operating points, and points that fell below the threshold were removed from the stack. This process continues until no grid points exceed the error threshold, yielding a set of operating points that provides a piecewise‐linear approximation of the model across the input space.

Network outputs are computed once for all grid points across the perturbation space and stored before the greedy search, and the Jacobian matrix is evaluated only when a new operating point is selected. Therefore, the computational cost for network evaluations is fixed for a predefined grid space, whereas the cost for Jacobian evaluations depends on the number of operating points required to approximate the network across those grid points.

A key limitation of this approach is its computational cost in high‐dimensional spaces, as the number of grid points grows exponentially with dimension. One potential solution is adaptive grid refinement, in which a coarse grid is iteratively refined in regions where local errors exceed the threshold. This concentrates computational effort in more nonlinear regions. This refinement strategy will be explored in future work.

### 2.2 Experimental methods

#### 2.2.1 Image data

In this study, we trained a CT denoising network using the RED‐CNN architecture.^15^ We used thoracic CT scans from the LIDC database,^16^ which contains 1018 distinct scans. For each scan, we selected five axial slices approximately evenly distributed through the longitudinal direction of the thorax. A total of 5090 slices were selected. All slices were resampled to a common voxel size of 0.9788 × 0.9788 mm, which is the largest in the LIDC dataset. The slices were converted from Hounsfield units to attenuation coefficients and divided by the approximate linear attenuation coefficient of bone (0.034 mm^−1^) to normalize the majority of voxel intensities to the range of 0 to 1. These slices served as the ground truth for simulating CT reconstructions.

Using the ground truth data, we simulated normal and low dose reconstructions for training and evaluation of the network. The simulations emulated a flat‐panel detector with a pixel size of 1.6 mm. The acquisition geometry was set to a source‐to‐axis distance (SAD) of 500 mm, a source‐to‐detector distance (SDD) of 1000 mm, and 720 view angles uniformly distributed over 360 degrees. A distance‐driven forward and back projector was employed. Independent Poisson noise was added to the projection data corresponding to specific barebeam fluence levels. Reconstructions were then performed using the filtered backprojection (FBP) algorithm with a Hamming window and a cutoff at the Nyquist frequency. The reconstruction voxel size was set to 0.8 mm in all directions. Projection and reconstruction were performed using the CTorch package.^17^ The nominal normal‐dose and low‐dose CT pairs used to train the network correspond to a barebeam fluence, $I_{0}$, of $4.8\times 10^{4}$ and $1.2\times 10^{4}$ photons per pixel (quarter dose reduction), respectively.

#### 2.2.2 Networks and training

The dataset was split into 80% for training, 5% for validation, and 15% for testing. During training and testing, we randomly selected five 55×55 patches within the thorax region of each slice. This was done to reduce computational burden, reduce overfitting to global structures, and generalize the network to different image sizes. The model was trained using a batch size of 10 and mean square error as the loss function. The optimizer was the Adam optimizer^18^ with $\beta_{1}$ set to 0.99 and $\beta_{2}$ set to 0.999. We trained the model for 400 epochs and used an initial learning rate of $10^{-4}$. We applied a step decay learning rate with a drop rate of 0.1 and a step size of 400. The model was trained using two RTX A4500 GPUs, with a total training of approximately two and a half hours. The validation mean square error stabilized after approximately 300 epochs, indicating that the model had converged.

While the strictly local linear region analysis only applies to networks with piecewise linear activation functions like ReLUs, the approximately local linear region analysis can be applied to differentiable architectures for which local Jacobians can be computed, subject to empirical validation for each architecture and input domain. To evaluate the generalizability of the proposed framework beyond the original RED‐CNN architecture, we performed additional analyses on two different denoising models.

First, we evaluated a RED‐CNN variant in which the ReLU activation function was replaced by GELU.^19^ All other training settings remained the same. This modification introduces a different nonlinear activation function while maintaining the overall convolutional architecture. The remaining training settings are the same as those for the RED‐CNN model.

Second, we evaluated a U‐ViT‐based denoising model that incorporates transformer blocks and global attention mechanisms.^20^ The network uses 5 × 5 patch embeddings with a 12‐layer transformer, 384‐dimensional embeddings, 6 attention heads, and a 4× MLP ratio. We used Adam optimizer with a 1e‐4 learning rate over 400 epochs. Compared with convolutional architectures, the transformer‐based model introduces global context modeling and a substantially different network structure. The remaining training settings are the same as those for the RED‐CNN model.

The denoising performance of all networks was evaluated across 1000 independent test patches of size 55×55 pixels using Peak Signal‐to‐Noise Ratio (PSNR), Structural Similarity Index Measure (SSIM), and Mean Squared Error (MSE). Furthermore, to evaluate the fidelity of the local linear approximation for the networks, we generated two independent noisy image realizations for each test patch, $\mu_{1}$ and $\mu_{2}$; we calculated the linear prediction as:

(13) $$\hat{H}\left(\mu_{2}\right)=H\left(\mu_{1}\right)+J_{\mu_{1}}\left(\mu_{2}-\mu_{1}\right),$$

where $J_{\mu_{1}}$ is the Jacobian of the network evaluated at image $\mu_{1}$.

#### 2.2.3 Strictly local linear regions

In this study, we focused only on perturbations of clinically relevant features (e.g., lesion contrast, lesion size, and background level). To investigate strictly local behavior when perturbing lesion contrast, we inserted a round lesion with a diameter of 9 mm into the slice shown in Figure 1. We adopted a noiseless simulation to focus on the perturbation of the input. We varied its contrast from 0 to 0.02 ${\mathrm{mm}}^{-1}$ while keeping track of each ReLU unit in the model. We also tested perturbations of varying lesion size, again tracking all changes in the activation pattern. In addition, we examined the 1D Jacobian profiles at example pixels across varying lesion contrasts to visualize differences between neighboring strictly local linear regions. As shown in Figure 1, we chose sample locations at the lesion center, three across the lesion edge, and one in the background.

**FIGURE 1:**
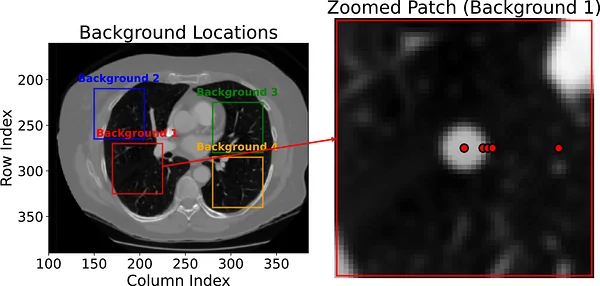
The selected slice with synthetic lesion insertion. The left image shows the full denoised CT slice with a red box indicating the lesion insertion region, which is 55×55 pixels in size. The left plot also shows the three other regions used for the background variation study. The right image provides a zoomed‐in view of the boxed region, showing the inserted lesion in greater detail. This patch was used for the experiments on local linear regions. The red dot represents the pixels used to visualize the 1D Jacobian.

#### 2.2.4 Approximately local linear regions

Approximate linearity was probed with two complementary analyses, both built on the local linear approximation error of Equation (12). In the first, a single operating point is fixed and the error is evaluated for a prescribed perturbation away from it, measuring how far one linear model can be carried. This is used for perturbations that cannot be placed on an ordered grid, namely the noise realization and the surrounding anatomy. In the second, the partitioning algorithm of Section 2.1.2 is applied over a gridded feature space to determine how many operating points are required to cover it. This is used for the lesion and background feature perturbations and for the comparison across dose reduction levels.

Several conventions are common to both. Rather than averaging Jacobians over multiple noise states, we computed the Jacobian at each operating point from a single noisy input image, while averaging the approximation error over R=50 independent noise realizations. The error was averaged over the central 25×25 subregion of the 55×55 input patch, so that the metric reflects the area containing the perturbed features. The average per‐pixel percentage error is defined as

(14) $$\bar{e}=\frac{1}{RM}\sum_{r=1}^{R}\sum_{i=1}^{M}\frac{|{\hat{H}}_{i}\left(\mu_{r}\right)-H_{i}\left(\mu_{r}\right)|}{|H_{i}\left(\mu_{r}\right)|}\times 100\%,$$

where $\mu_{r}$ is the r‐th noisy perturbed input, M is the total number of pixels in the central 25×25 subregion, and $H_{i}$ and ${\hat{H}}_{i}$ are the i‐th pixel of the network output and its linear approximation, respectively. A threshold of $\bar{e}=5\%$ was used throughout. Because the Jacobian comes from one noise realization, the reported error captures the joint effect of the feature perturbation and the noise fluctuation; the contribution of the noise alone is isolated first and serves as a reference level for the analyses that follow.

For the partitioning analyses, a 30×30 grid was placed over each two‐dimensional input space. As a reference for computational cost, the size–contrast experiment required approximately 53 minutes on a single NVIDIA RTX A4500 GPU, with 3 operating points selected by the algorithm. The proposed method is intended for offline analysis of low‐dimensional, clinically specified feature spaces, rather than for real‐time inference or high‐dimensional input spaces, where the number of grid evaluations grows exponentially with dimensionality. Within this intended scope, the processing time poses no practical limitation. Unless otherwise stated, analyses were performed on the RED‐CNN network trained at quarter dose; the same partitioning was also applied to the RED‐CNN‐GELU variant and the U‐ViT architecture to evaluate how the activation function and the underlying network connectivity affect the number and boundaries of the approximately local linear regions.

##### 2.2.4.1 Noise realizations

Because input noise inherently acts as a perturbation, this experiment specifically investigates whether the network remains approximately linear across different noise realizations and how this stability depends on the degree of noise reduction. To isolate the effect of noise, we evaluated the Jacobian of the RED‐CNN network at an initial realization $\mu_{0}$ across three dose reduction levels (1/2, 1/4, and 1/8 dose) for a 9 mm lesion with a contrast of 0.015 mm^−1^. The predicted network response across 50 independent noise realizations $\mu_{i}$ is:

(15) $$\hat{H}\left(\mu_{i}\right)=H\left(\mu_{0}\right)+J_{\mu_{0}}\left(\mu_{i}-\mu_{0}\right).$$

The prediction was compared against the true output $H(\mu_{i})$ for each dose reduction, using a RED‐CNN, RED‐CNN‐GELU, and U‐ViT models trained specifically for that dose reduction factor.

##### 2.2.4.2 Lesion features and background intensity perturbations

The partitioning algorithm was evaluated across three distinct two‐dimensional (2D) feature spaces, pairing lesion contrast ($0.005\;{\text{mm}}^{-1}$ to $0.08\;{\text{mm}}^{-1}$) with: (1) background intensity ($0\;{\text{mm}}^{-1}$ to $0.035\;{\text{mm}}^{-1}$), applied uniformly over the 55×55 pixel region); (2) lesion size (diameter from 6mm to 12mm); and (3) spicule length. For the spiculation experiment, a circular lesion was modified by adding eight evenly spaced radial spikes with lengths ranging from 0.9mm to 9mm (10% to 100% of the lesion diameter), modeled as narrow triangular extensions. In each 2D space, non‐perturbed parameters were held constant at baseline values: a 9mm lesion diameter, a background intensity of $0.001\;{\text{mm}}^{-1}$, and no spiculation. Figure 2 shows examples of these resulting feature variations.

**FIGURE 2:**
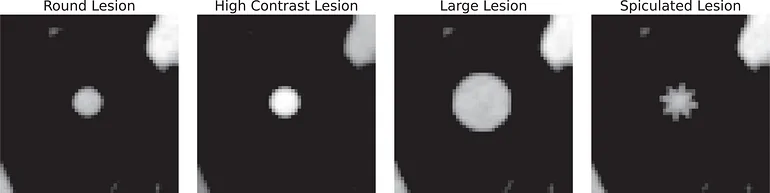
Demonstration of lesion variations used in the partitioning algorithm experiments, from left to right: baseline round lesion (6 mm diameter with 0.015 ${\mathrm{mm}}^{-1}$ contrast), high‐contrast round lesion (0.02 ${\mathrm{mm}}^{-1}$ contrast), enlarged round lesion (9 mm diameter), and spiculated lesion with radial spikes (3 mm spike).

We additionally performed a three‐dimensional perturbation experiment over lesion contrast, lesion size, and background intensity using a 20×20×20 grid. The ranges were: contrast from 0.005 to 0.08 ${\mathrm{mm}}^{-1}$, diameter from 6 to 12 mm and background intensity from 0.001 to 0.035 ${\mathrm{mm}}^{-1}$.

##### 2.2.4.3 Anatomical background variation

This experiment specifically evaluates whether the network's local linear behavior generalizes across different anatomical background regions. Because background anatomy changes categorically from one region to another rather than varying continuously along an axis, it cannot be parameterized into the grid‐based partitioning algorithm and is therefore evaluated separately. We used the four background ROIs shown in Figure 1, all drawn from lung but differing in the surrounding airway structures. Let $\mu_{s}$ denote a lesion‐containing image formed by inserting the lesion into the background region s∈{2,3,4}, and let $\mu_{1}$ denote the corresponding image in target Region 1. For each source region in turn, we computed the Jacobian at $\mu_{s}$ and used it to predict the network response at $\mu_{1}$:

(16) $$\hat{H}\left(\mu_{1}\right)=H\left(\mu_{s}\right)+J_{\mu_{s}}\left(\mu_{1}-\mu_{s}\right).$$

Three lesion configurations were evaluated: (i) 6 mm diameter with 0.015 ${\mathrm{mm}}^{-1}$ contrast, (ii) 9 mm diameter with 0.015 ${\mathrm{mm}}^{-1}$ contrast, and (iii) 6 mm diameter with 0.020 ${\mathrm{mm}}^{-1}$ contrast. Because the perturbation $\mu_{1}-\mu_{s}$ contains both a background change and a noise change, the resulting prediction error is not attributable to anatomy alone. The noise‐only error, obtained by evaluating the same prediction across independent noise realizations within Region 1 for each of the three lesion configurations, is therefore reported alongside it so that the additional error introduced by the change of anatomy can be assessed from the difference between the two.

Finally, we tested whether the surrounding anatomy alters the partition boundaries themselves. We repeated the partitioning analysis over the same three two‐dimensional input spaces on the Region 1 dataset, but constructed the linear approximation from Jacobians computed at Region 4 under an identical lesion‐insertion protocol, allowing direct comparison against the within‐region partitions.

##### 2.2.4.4 Dose reduction level

Dose reduction increases noise, which may amplify network nonlinearity. The nominal network was trained on pairs in which the low‐dose images were reduced to a quarter of the normal dose; networks trained at other reduction levels, especially more aggressive ones, may exhibit different degrees of linearity. To quantify this, we trained two additional models using the same normal‐dose CT but with the low‐dose CT changed to one half ($I_{0}=2.4\times 10^{4}$) and one‐eighth ($I_{0}=0.6\times 10^{4}$) of the normal dose. For each, we repeated the two‐dimensional partitioning. In addition, we visualized the two‐dimensional Jacobian profiles at the center of a circular lesion to assess how dose reduction influences the local response characteristics of the network.

## 3 RESULTS

### 3.1 Model denoising performance

The statistical summary of the denoising performance and the corresponding improvements over the low‐dose input are detailed in Table 1. Both the convolutional and transformer‐based networks achieved the expected noise reduction while preserving critical structural details. The primary RED‐CNN model improved the average PSNR from 40.72 dB to 45.46 dB and increased the SSIM from 0.9171 to 0.9721. The RED‐CNN‐GELU variant demonstrated highly comparable denoising efficacy, yielding a final PSNR of 45.18 dB and an SSIM of 0.9712. In contrast, the transformer‐based U‐ViT model achieved a slightly lower baseline denoising performance with a post‐processed PSNR of 43.78 dB and an SSIM of 0.9541, while still substantially reducing the mean squared error relative to the low‐dose input.

**TABLE 1:** Quantitative denoising performance comparison and cross‐noise linear approximation fidelity $\hat{H}\left(\mu_{2}\right)$ across 1000 test patches (Mean ± standard deviation).

Model / Evaluation	PSNR (dB)	SSIM	MSE $\times 10^{3}$
Low‐dose baseline	40.72±3.12	0.9171±0.0598	0.1153±0.1281
RED‐CNN	45.46±3.78	0.9721±0.0236	0.0429±0.0520
Linear approx	45.27±3.80	0.9703±0.0254	0.0451±0.0561
RED‐CNN‐GELU	45.18±3.84	0.9712±0.0250	0.0464±0.0584
Linear approx	45.12±3.85	0.9705±0.0254	0.0472±0.0601
U‐ViT	43.78±4.09	0.9541±0.0264	0.0645±0.0705
Linear approx	43.75±4.06	0.9529±0.0268	0.0642±0.0668

*Note*: MSE values are scaled by $10^{3}$.

As detailed in Table 1, the linear approximation $\hat{H}\left(\mu_{2}\right)$ closely tracks the non‐linear network response across all architectures, yielding nearly identical PSNR, SSIM, and MSE metrics. This demonstrates that the local linear approximation is accurate for 1/4‐dose‐level noise fluctuations.

To visually assess these localized denoising characteristics, Figure 3 illustrates representative denoising results. The example compares the ground‐truth target structures, the simulated noisy low‐dose inputs, and the final filtered outputs across the three evaluated model configurations. In addition, we show the corresponding linear approximation result of each model using an independent simulated noise realization.

**FIGURE 3:**
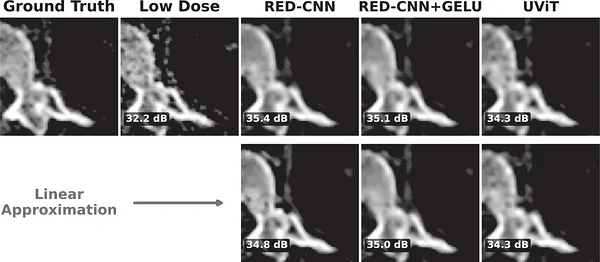
Example denoising results for different network architectures with their linear approximation in the second row. From left to right: ground truth image, low‐dose input, RED‐CNN, RED‐CNN‐GELU, and U‐ViT.

### 3.2 Strictly local linear region

We investigated the sensitivity of the activation patterns of the network to small perturbations in lesion contrast and size. Figures 4 and 5 illustrate the number of cumulative changes in activation patterns from the signal‐absent background as a function of lesion contrast and size, respectively, along with zoomed views highlighting regions where activation patterns remain the same over a finite range of inputs. For reference, the total number of ReLU units in RED‐CNN is around 1.9 million. Over the entire range of perturbations, a very large number of ReLU changes were observed. When zoomed in to a fine range of perturbations, we observed that multiple ReLU units often change their activation states even with fine perturbation steps, indicating frequent transitions between local linear regions. Nevertheless, there exist segments where the activation pattern remains unchanged across multiple steps, corresponding to strictly local linear regions.

**FIGURE 4:**
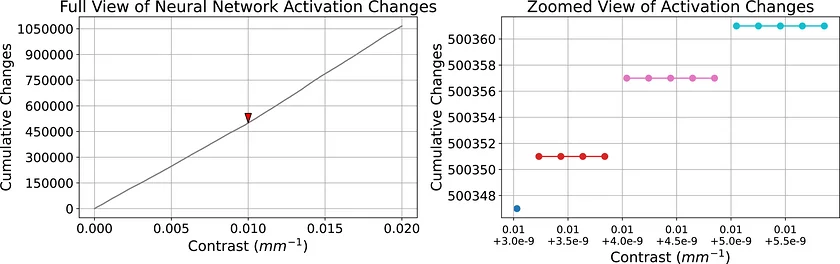
Cumulative changes in activation patterns, computed via Equation (10), as a function of lesion contrast (left). The right panel shows a zoomed region where the model exhibits strictly local linear behavior across multiple contrast steps.

**FIGURE 5:**
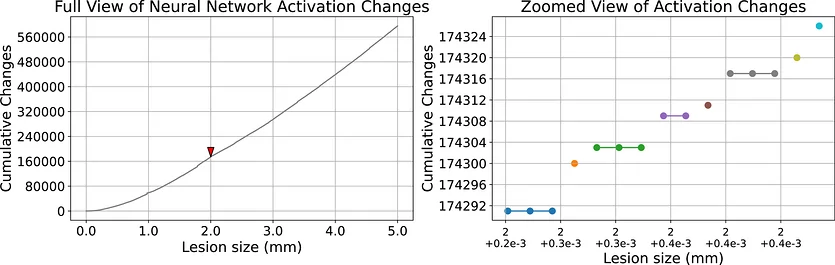
Cumulative changes in activation patterns, computed via Equation (10), as a function of lesion size (left). The right panel shows a zoomed region where the model exhibits strictly local linear behavior across multiple size steps.

Although the strictly local linear region only covers a small portion of the input space and a large number of cumulative changes in activation states were observed, the local system responses (defined by the Jacobian) in these regions are highly similar over a relatively large range of perturbations. Figure 6 compares the one‐dimensional Jacobian profiles at five representative pixel locations shown in the zoomed view of Figure 1, including one at the lesion center, three along the lesion boundary, and one in the background. Each plot shows the Jacobian at five different lesion contrasts ranging from 0.01 to 0.018 ${\mathrm{mm}}^{-1}$. Here, the one‐dimensional Jacobian denotes the one‐dimensional profile through the full two‐dimensional Jacobian at the selected output pixel location, taken along the horizontal axis containing the output pixel of interest. At each location, the Jacobians are highly consistent across this contrast range despite corresponding to distinct activation patterns, indicating that the network response can be well approximated as linear beyond strictly local linear regions. Jacobians show greater variations spatially, indicating shift‐variant system responses.

**FIGURE 6:**
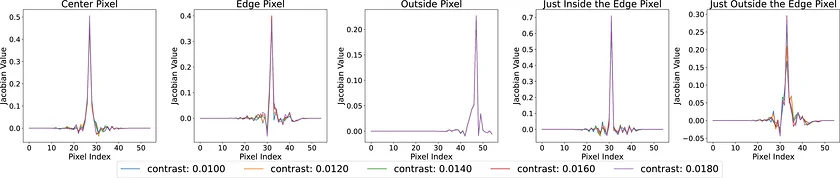
One‐dimensional Jacobian profiles at the lesion center, around the lesion edge, and at background pixels across varying lesion contrasts (0.01–0.018 ${\mathrm{mm}}^{-1}$). Profiles demonstrate consistent Jacobian behavior across lesion contrast, indicating approximate linearity. Spatial variations in Jacobians are observed, indicating shift‐variant system responses.

### 3.3 Approximately local linear regions

Approximate linearity was assessed with the two analyses introduced in Section 2.2.4. We first report the transfer error for perturbations for the noise realization, which together establish the baseline error against which the gridded results are read. We then report the partitioning results over the lesion and background feature spaces. We then report results for background anatomical variation and across dose‐reduction levels.

#### 3.3.1 Noise realizations

We quantified the approximation error attributable to the noise alone, and examined how this error changes as the dose reduction factor increases. The resulting errors across the 50 noise realizations at each dose level are summarized in Table 2 for the RED‐CNN, RED‐CNN‐GELU, and U‐ViT models, which reports the mean and standard deviation of the per‐pixel percentage error.

**TABLE 2:** Noise‐only local linear approximation errors across 50 independent noise realizations for each network at three dose reduction levels.

Model	1/2 dose	1/4 dose	1/8 dose
RED‐CNN	2.54%±0.21%	2.96%±0.22%	4.31%±0.30%
RED‐CNN‐GELU	0.46%±0.05%	0.74%±0.07%	2.14%±0.20%
U‐ViT	0.21%±0.02%	0.44%±0.04%	0.96%±0.13%

*Note*: Values are mean ± standard deviation of the per‐pixel percentage error.

For all three networks, the noise‐only error increases systematically with higher levels of dose reduction (i.e., increased noise levels). For the RED‐CNN, the error increases from 2.54% at 1/2 dose to 2.96% at 1/4 dose, and reaches 4.31% at 1/8 dose. Across all networks and dose levels, the noise‐only error remains below the 5% threshold used to define approximately local linear regions, indicating that evaluating J at a single noise realization provides a reasonable approximation. The approximation error reported in later sections therefore reflects both noise and lesion perturbations rather than lesion features alone. For the RED‐CNN at 1/8 dose, the noise contribution alone approaches this threshold, leaving little margin before it dominates the linear approximation error, consistent with the finer partitioning observed at 1/8 dose in later sections.

Notably, the RED‐CNN‐GELU and U‐ViT models exhibit substantially lower noise‐only errors than the ReLU‐based RED‐CNN, indicating that the RED‐CNN exhibits a higher degree of nonlinearity with respect to noise perturbations. However, this does not imply that the RED‐CNN‐GELU and U‐ViT models require fewer approximately local linear regions in feature space, where larger, structured perturbations dominate the approximation error.

#### 3.3.2 Lesion features and background intensity perturbations

To assess model linearity over a varying input feature space, we first evaluated the approximation error when using only a single central operating point, on the size‐versus‐contrast and background‐versus‐contrast parameter spaces. Figure 7a,b shows the average pixel approximation error across these grids. In the background‐versus‐contrast plot, errors exceeded 5% in certain regions, while in the size‐versus‐contrast plot, errors were high across a larger portion of the input space. These results highlight the limitations of a single‐point linear approximation and motivate the use of a piecewise linear approach.

**FIGURE 7:**
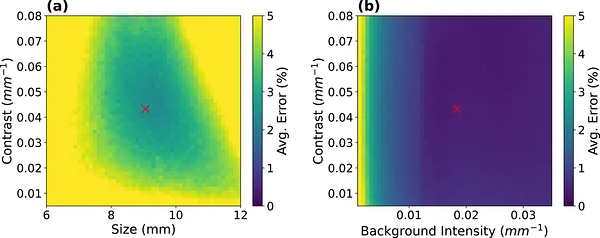
Assessment of linear approximation accuracy using a single operating point. (a)–(b) Average pixel‐wise approximation error across the input space for contrast–size and contrast–background perturbations, respectively. The approximation error increases with distance from the operating point, and similar trends are observed across different clinically relevant input dimensions.

We therefore applied the greedy grid search algorithm to partition the input space into approximately local linear regions by identifying a minimal set of operating points. Under the 5% approximation error threshold, the algorithm required only 3–4 operating points to achieve full coverage of the investigated parameter spaces.

Figure 8a–c shows the resulting partitions for the contrast–background, contrast–size, and contrast–spiculation spaces, respectively. In these figures, each color denotes a distinct approximately local linear region, and the corresponding operating point within each region is indicated by a highlighted X marker. Figure 8d–f shows the local approximation error evaluated using the Jacobian at the assigned operating point for all points in the partitioned input space.

**FIGURE 8:**
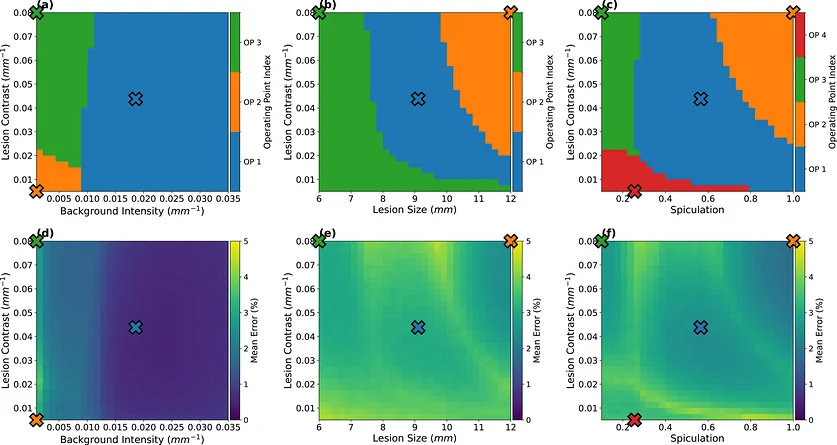
Greedy partitioning of the input space into approximately local linear regions. Panels (a)–(c) show the best approximating operating point assigned to each grid location in the contrast–background intensity, contrast–lesion size, and contrast–spiculation scale spaces, respectively, with colors indicating distinct approximately local linear regions. Panels (d)–(f) show the corresponding average approximation error (%) obtained when the model output is locally approximated using the assigned operating point for the same parameter spaces.

As shown in these figures, the greedy algorithm, by design, tends to place new operating points at grid locations that are furthest from existing operating points and that exhibit the largest approximation errors, often near the boundaries or extremes of the input space. This behavior ensures efficient coverage of regions where the network response deviates most from existing linear approximations. Once an operating point is added, surrounding regions are absorbed into the same approximately local linear region until the error threshold is met.

For the three‐dimensional perturbation space with contrast, lesion size, and background intensity varying, the greedy grid search identified only four operating points as sufficient to approximate the network response across the entire input space under the predefined error threshold. Figure 9 visualizes the selected operating points and the corresponding partitioning of the input space, where each grid point is assigned to the operating point that provides the best local linear approximation. These results indicate that, for modest and structured perturbations, the network response is well described by a small number of locally linear regions.

**FIGURE 9:**
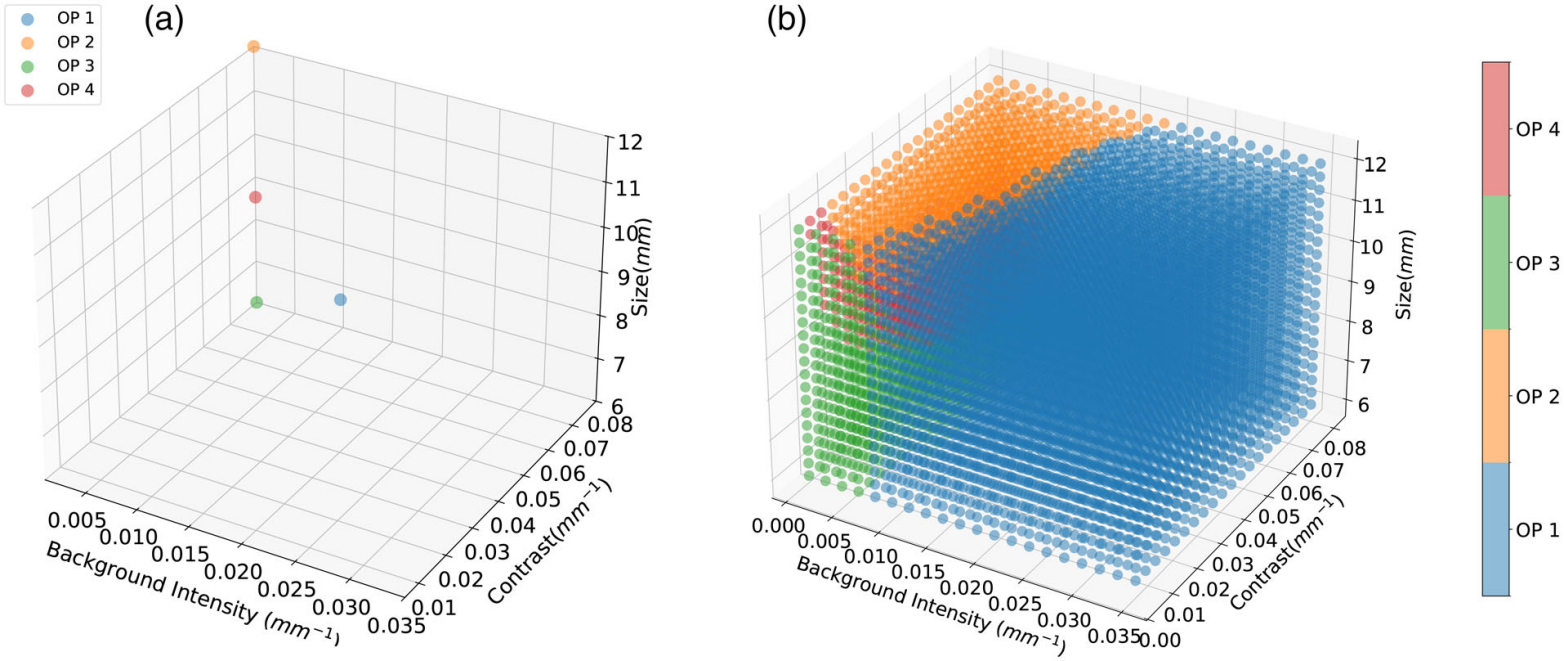
Three‐dimensional analysis of operating point selection in the contrast–size–background intensity space. (a) scatter plot of the operating points identified by the greedy algorithm. (b) assignment of each grid point in the input space to its best approximating operating point. Together, the panels illustrate that only a small number of operating points are required to achieve accurate piecewise linear approximation across the full 3D parameter space.

To evaluate how network architecture impacts local linearity, we extended this partitioning analysis to the RED‐CNN‐GELU and U‐ViT models across the same input spaces (Figure 10). The RED‐CNN‐GELU model exhibited a highly similar structural landscape to the original RED‐CNN, requiring a comparable number of operating points to satisfy the error threshold. This suggests that transitioning from a piecewise‐linear activation function (ReLU) to a smooth, closely matched non‐linear counterpart (GELU) does not radically alter the macro‐scale local linearity of the convolutional network. In contrast, the transformer‐based U‐ViT model displayed significantly stronger non‐linear behavior across all tested features. Due to its complex global attention mechanisms, the U‐ViT model required a substantially larger number of operating points to achieve the same approximation error threshold, resulting in a denser and more fragmented partition map.

**FIGURE 10:**
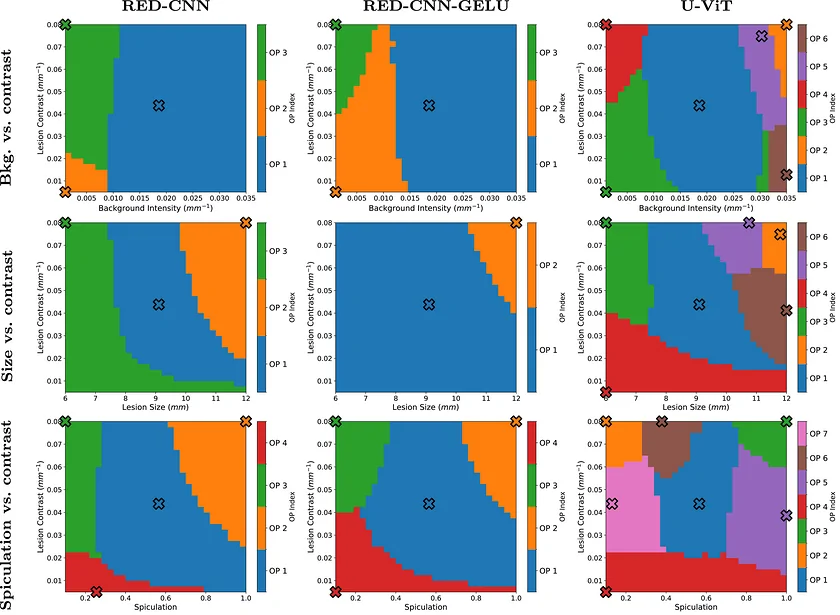
Comparison of operating points across different models. Abbreviation: Bkg., Background.

Together, these results demonstrate that a relatively small number of operating points remains sufficient to construct accurate piecewise‐linear approximations across clinically relevant variations in contrast, lesion size, and background intensity. This efficiency highlights the practical feasibility of applying linear system analysis to characterize and interpret complex, highly non‐linear deep learning models.

#### 3.3.3 Anatomical background variation

To evaluate whether the network's local linear behavior holds across different anatomical contexts, we evaluated the cross‐region linear approximation error using the four distinct background ROIs shown in Figure 1. Table 3 summarizes the cross‐region prediction errors when Jacobians evaluated at Regions 2, 3, or 4 are used to approximate the network response at Region 1, alongside the noise‐only baseline reference with Jacobians evaluated at Region 1.

**TABLE 3:** Cross‐region local linear approximation errors (mean ± std of the per‐pixel percentage error) for predicting Region 1 using Jacobians evaluated at Regions 2–4.

Jacobian source	6 mm, 0.015 mm^−1^	9 mm, 0.015 mm^−1^	6 mm, 0.020 mm^−1^
Reference	3.00%±0.22%	2.96%±0.22%	3.04%±0.22%
Region 2	5.89%±0.27%	5.17%±0.22%	5.83%±0.26%
Region 3	4.78%±0.23%	4.63%±0.25%	4.84%±0.23%
Region 4	3.66%±0.19%	3.45%±0.18%	3.78%±0.21%

*Note*: Reference represents the noise‐only baseline error with Jacobians evaluated at Region 1.

Transferring the Jacobian across background regions increases the central‐region mean percentage error above baseline in all cases: by roughly 0.6% for Region 4, 1.8% for Region 3, and 2.6% for Region 2. This error is governed primarily by the source anatomy rather than the specific lesion configuration. Regions 3 and 4 maintain errors below the 5% threshold for all configurations, whereas Region 2 exceeds this threshold in every configuration.

Thus, the additional error from background variation grows systematically with the dissimilarity of the surrounding anatomy. This suggests that the local response to the target lesion itself, which dominates the local image error, is relatively stable, whereas the response to the surrounding background structures imposes practical limits on cross‐background generalization.

Finally, we evaluated whether the partition boundaries themselves change significantly if the two background regions are similar. Repeating the partitioning analysis on Region 1 using Jacobians evaluated at Region 4 yielded the partition layouts and error dynamics shown in Figure 11. The partition configurations and total number of required operating points closely mirror those in Figure 8, exhibiting only minor spatial variations at the boundaries. Therefore, Jacobian transfer between two similar regions introduces an overall shift in baseline error without altering the underlying piecewise‐linear partition structure.

**FIGURE 11:**
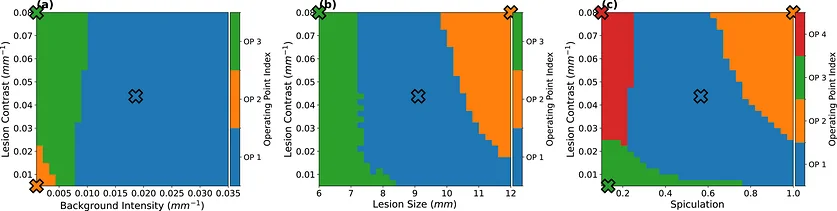
Partitioning results for Region 1 using Jacobians evaluated at background Region 4. Panels (a)–(c) map the assigned operating points across (a) contrast versus background intensity, (b) contrast versus lesion size, and (c) contrast versus spiculation scale spaces. The resulting partition layouts closely match Figure 8, demonstrating structural stability under varying lung background contexts.

#### 3.3.4 Dose reduction level

The effect of dose reduction level on the number of approximately local linear regions is shown in Figure 12. Results from the partition algorithm for three different input feature spaces (rows) are shown for three networks trained at different dose reduction levels (columns). We observed that models trained at lower dose levels required a greater number of operating points to satisfy the predefined error threshold, indicating an increased degree of nonlinearity under these conditions. This trend is consistent with the intuition that denoising networks operating at lower dose levels must perform more complex mappings to suppress stronger noise while preserving anatomical structures.

**FIGURE 12:**
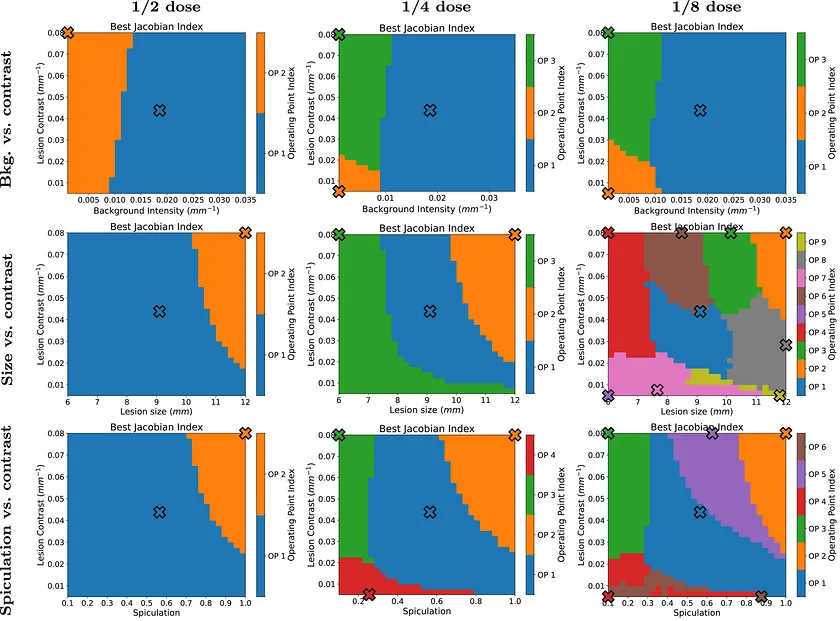
Comparison of operating points across models trained at different dose levels. Abbreviation: Bkg. (Background).

Notably, in the spiculation‐versus‐contrast experiment at one‐eighth dose, a subset of grid points remained above the error threshold even after being selected as operating points, so no partition satisfying the prescribed tolerance could be formed over the full space. This is consistent with the noise‐only error of Section 3.3.1: the combination of high noise levels and complex lesion features such as spiculation induces sufficiently strong nonlinearities, indicate the network is highly nonlinear within this feature space.

We further examined the two‐dimensional (2D) Jacobian at the lesion center to assess how dose reduction levels in the training data influence the response of the network. Figure 13 shows the 2D Jacobian of the output pixel at the center of the lesion with respect to all pixels in the input patch. Jacobians for models trained using lower dose data are broader, indicating stronger spatial smoothing in response to increased noise.

**FIGURE 13:**
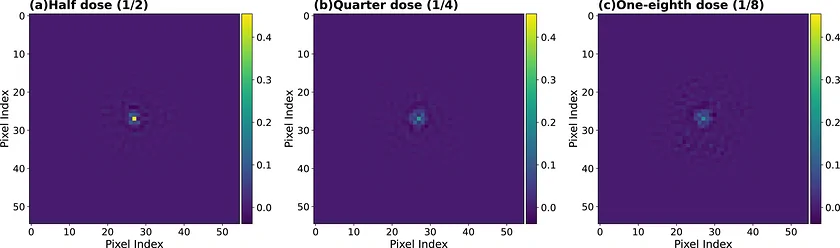
Two‐dimensional Jacobian profiles at the lesion center for models trained at different dose levels. (a) 1/2 dose, (b) 1/4 dose, and (c) 1/8 dose. Each panel shows the response of all image pixels with respect to the center pixel. Lower‐dose models exhibit broader Jacobian profiles, reflecting stronger smoothing applied by the network to compensate for increased noise.

## 4 DISCUSSION

In this study, we investigated the locally linear behavior of neural networks for CT denoising. While tracing ReLU activation patterns showed that strictly local linear regions are extremely small, with many units toggling state over perturbations as small as 0.02 ${\mathrm{mm}}^{-1}$ in lesion contrast, the underlying system responses (Jacobians) remained remarkably similar. This demonstrates that the network can be approximated as locally linear over broader regions of the feature space.

Before partitioning the feature spaces, we quantified the contribution of the noise realization itself to the approximation error, since it acts as a perturbation as well. For the RED‐CNN, this noise‐only error remained below the 5% threshold at half and quarter dose, but rose to 4.3% at one‐eighth dose, approaching the threshold, indicating that the network becomes more nonlinear with respect to noise as the noise level increases. The same trend occurs for RED‐CNN‐GELU and UViT as well. This trend anticipates the dose‐dependent behavior discussed below.

By grouping regions with similar Jacobians under a predefined error threshold, we partitioned clinically relevant input spaces over lesion size, contrast, spiculation, and background into just a few operating points. Networks trained with more aggressive dose reduction required substantially more regions over the same input space, and at one‐eighth dose, a subset of grid points in the spiculation–contrast space remained above the error threshold even after being selected as operating points, so no partition satisfying the prescribed tolerance could be constructed. This is not a failure of the partitioning algorithm, but rather reflects that the combination of higher noise and complex lesion morphology pushes the network into a more strongly nonlinear regime at this dose level.

We further examined how the surrounding anatomical background affects the local response, transferring Jacobians between four lung regions with distinct airway structures. Transfer increased the central‐region error above the noise‐only baseline in every case, with the magnitude of the increase tracking the dissimilarity of the source and target anatomy. This indicates that the network's response to the lesion itself is comparatively stable, while its response to surrounding structure imposes a practical limit on how broadly a single Jacobian can be reused across anatomical contexts.

While demonstrated primarily on a standard ReLU CNN, the approximately local linear region analysis was applied directly to the GELU‐based RED‐CNN and U‐ViT for 1/4 dose reduction. GELU‐based RED‐CNN requires a similar number of operating points, while U‐ViT requires significantly more operating points. This hints that U‐ViT exhibits a higher degree of nonlinearity.

The primary objective of this study was to establish an interpretability framework by characterizing network mechanics, and a crucial next step is validating how these linearity properties translate to clinical efficacy. Future work will systematically investigate the direct relationship between a network's local linearity profile and its performance on specific downstream clinical tasks, such as task‐based lesion detectability, segmentation robustness, and the reproducibility of radiomics features. These investigations will be pursued in future studies.

## 5 CONCLUSION

In this study, we investigated the locally linear behavior of an example neural network for CT denoising by analyzing strictly and approximately local linear regions within specific input spaces. Our findings demonstrate that while activation patterns are highly sensitive to small input perturbations, resulting in strictly local linear regions that occupy only a small region of the input space, the Jacobians remain remarkably similar across a much larger range of perturbations. This observation suggests the presence of a broader approximately local linear region, enabling the system to be effectively described using a small set of strategically chosen operating points. Results in this work show that local linear analysis can be a useful way to interpret and approximate “black‐box” style neural networks in medical imaging.

## CONFLICT OF INTEREST STATEMENT

Craig Abbey serves as an advisor to Izotropic Corp. LLC and as an occasional consultant to Canon Medical Research Corp. J. Webster Stayman reports academic‐industry partnerships involving direct funding from Canon Medical Research, EOS Imaging, Fischer Imaging, and Siemens Healthineers. He maintains a research partnership with OsteoSys involving government‐funded research, and partnerships with Varex and Hamamatsu involving no‐cost equipment loans. Grace J. Gang reports an academic‐industry partnership with Siemens Healthineers.
